# Role of Cellular Lipids in Positive-Sense RNA Virus Replication Complex Assembly and Function

**DOI:** 10.3390/v2051055

**Published:** 2010-04-29

**Authors:** Kenneth A. Stapleford, David J. Miller

**Affiliations:** 1 Section of Microbial Pathogenesis, Yale University School of Medicine, New Haven, CT 06519, USA; E-Mail: kenneth.stapleford@yale.edu; 2 Department of Internal Medicine, University of Michigan Medical School, Ann Arbor, MI 48109, USA; 3 Department of Microbiology & Immunology, University of Michigan Medical School, Ann Arbor, MI 48109, USA

**Keywords:** RNA viruses, replication, membranes, lipids

## Abstract

Positive-sense RNA viruses are responsible for frequent and often devastating diseases in humans, animals, and plants. However, the development of effective vaccines and anti-viral therapies targeted towards these pathogens has been hindered by an incomplete understanding of the molecular mechanisms involved in viral replication. One common feature of all positive-sense RNA viruses is the manipulation of host intracellular membranes for the assembly of functional viral RNA replication complexes. This review will discuss the interplay between cellular membranes and positive-sense RNA virus replication, and will focus specifically on the potential structural and functional roles for cellular lipids in this process.

## 1. Introduction

The virosphere is large, complex, and continually expanding. One group of viruses responsible for a wide range of diseases in humans, animals, and plants are classified as positive-sense RNA viruses due to their genome structure, which consists of one or more single-stranded RNA molecules that in many respects resemble cellular mRNAs. Clinically important members of this group cause significant morbidity and mortality, include viruses from the *Picornaviridae*, *Flaviviridae*, *Caliciviridae*, *Coronaviridae*, and *Togaviridae* families, and represent a prominent component of the growing list of emerging and potentially devastating health threats [1,2]. Currently approved therapies for infections with these pathogens are limited, and the development of specific viral enzyme-targeted inhibitors is frequently complicated by the inherently high mutation rate of viral RNA polymerases and the rapid development of resistance [3,4]. An alternative approach that has been advocated in the development of novel and potential broadly active antivirals is the targeting of host processes, which range from blockade of cell surface receptors to altering cellular metabolism [5–9]. However, the cell-centric approach to antiviral development requires substantial knowledge and understanding of the host-pathogen interactions that control virus replication.

**Figure 1.**
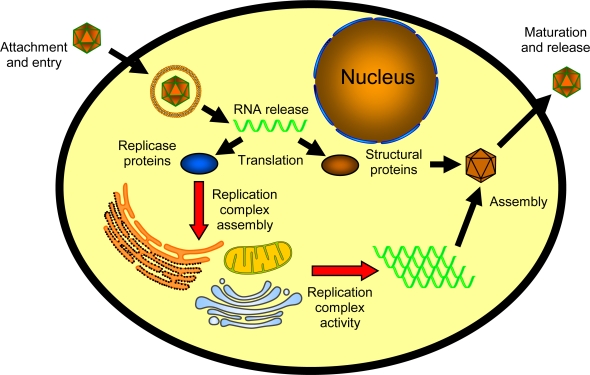
Schematic of positive-sense RNA virus replication cycle. General steps include: (i) attachment and entry; (ii) release of genome into cytoplasm; (iii) translation of genomic viral RNA into replicase or structural proteins; (iv) assembly of replication complex on host intracellular membrane; (v) amplification of viral genome via dsRNA intermediate; (vi) genome encapsidation; and (vii) maturation and release.

The small genomes of viruses relative to other organisms requires that they appropriate cellular machinery to complete their replication cycle, which for positive-sense RNA viruses is depicted schematically in Figure 1. For example, no virus encodes the complete set of nucleic acid and protein constituents necessary for autonomous translation of viral RNAs, which represents an important initial step in virus replication, and therefore positive-sense RNA viruses utilize diverse and often elaborate mechanisms to subvert the cellular translation apparatus to their benefit [10,11]. Another step in the life cycle of positive-sense RNA viruses that highlights the importance of cell components and virus-host interactions is the requirement of host-derived intracellular membranes for RNA replication [12–14]. This requirement is completely independent of their structural role during the encapsidation and assembly of enveloped viruses. The conclusion that cellular membranes are essential host factors in viral RNA replication is based primarily on four sets of observations. First, positive-sense RNA virus replication is associated with dramatic intracellular membrane rearrangements, which are readily demonstrated by electron microscopy [15–39]. Second, viral proteins with known or hypothesized enzymatic activity linked to genome amplification, which are referred to collectively as replicase proteins, co-partition with intracellular membrane fractions. Furthermore, these membrane fractions retain viral replicase enzymatic activity that can be measured *in vitro* [18,26,28,40–45]. Third, detergents can disrupt, whereas phospholipids can enhance, *in vitro* viral replicase activity [28,42–45]. And fourth, pharmacologic or genetic disruption of lipid metabolism has been shown to modulate positive-sense RNA virus replication [46–63].

Although the observations outlined above provide substantial validation for the important role that cellular membranes play in positive-sense RNA virus replication, they provide only circumstantial evidence for the specific membrane components involved in the process and their precise molecular functions with respect to viral genome replication. Intracellular membranes contain diverse protein and lipid constituents and play a variety of roles during normal cellular physiology and metabolism, which include facilitating the spatial separation of cellular processes and the consequent differential concentrations of crucial cellular components, providing structural integrity to maintain organelle shape, and contributing functional co-factors for multiple processes such as signal transduction and biosynthesis. In this review, we will discuss three potential roles that lipid constituents of host cell membranes play in positive-sense RNA virus genome replication. These include: (i) providing a scaffold for targeting and assembly of RNA replication complexes; (ii) inducing alterations in membranes structure to potentially shield viral RNA replication intermediates from cellular innate antiviral pathways; and (iii) serving as functional co-factors for optimal enzymatic activity of viral replicase proteins. We will use examples of plant, insect, and mammalian viruses to highlight specific aspects of these potential roles, and will focus primarily on lipids rather than membranes in general. Several excellent reviews have recently been published [12–14] for readers interested in further exploring the connections between positive-sense RNA viruses and cellular membranes. In addition, readers with a particular interest in hepatitis C virus, one of the most clinically relevant positive-sense RNA viruses whose connection with cellular lipid metabolism is becoming increasingly apparent, are encouraged to explore the companion article by McLauchlan *et al.* forming part of this special issue of *Viruses*.

### 1.1. Lipids and replication complex targeting and assembly

The interior of a cell is a highly organized environment, where membrane-bound organelles and specific organelle membranes represent one mechanism whereby cells enforce spatial constraints on particular metabolic processes. For example, the enzymes involved in electron transport and respiration are concentrated on the inner mitochondria membrane. Similarly, viruses may use intracellular membranes as simple molecular scaffolds on which to assemble their replication complexes, thereby increasing replication efficiency by concentrating essential viral and potential cellular components within a smaller microenvironment in the cell. Furthermore, membranes may provide a convenient platform to coordinate various steps in the replication cycle (see Figure 1), such as the demonstrated coupling of viral RNA replication, translation, and genome packaging [64–66]. However, although positive-sense RNA virus replication complexes have been found associated with cellular membranes derived from a number of intracellular organelles, including the endoplasmic reticulum, Golgi apparatus, endosomes, lysosomes, peroxisomes, chloroplasts, and the mitochondria (reviewed in ref [12–14]), individual viruses show a particular membrane selectivity (Table 1). This selectivity suggests a specific “receptor•ligand”-type interaction between viral replicase proteins and intracellular membrane components. Several membrane-specific targeting signals within individual virus-encoded replicase proteins have been characterized in detail [26,67–70], but only a limited number of cellular membrane components have been shown to be important in viral RNA replication complex targeting and assembly, most of which are membrane proteins [71–73]. However, preferential interactions between positive-sense RNA virus replicase proteins and membrane phospholipids have been described [44,74], suggesting that lipids may also contribute to replication complex targeting and assembly. We recently demonstrated that the membrane-targeting viral replicase protein of Flock House virus, a model alphanodavirus that assembles its replication complexes on outer mitochondrial membranes [25], preferentially interacts with anionic phospholipids, and in particular cardiolipin [74]. Although most phospholipids are widely distributed throughout intracellular membranes, cardiolipin is found predominantly, if not exclusively, in mitochondrial membranes [75,76], suggesting a potential role for this particular lipid in Flock House virus RNA replication complex targeting and assembly. The ubiquitous nature of cellular phospholipids and the substantial technical challenges associated with studying lipid-membrane protein interactions at the molecular and structural levels has thus far hampered our ability to more fully examine the potential role that cellular lipids play in positive-sense RNA virus replication complex assembly.

### 1.2. Lipids and membrane structure alterations

**Figure 2.**
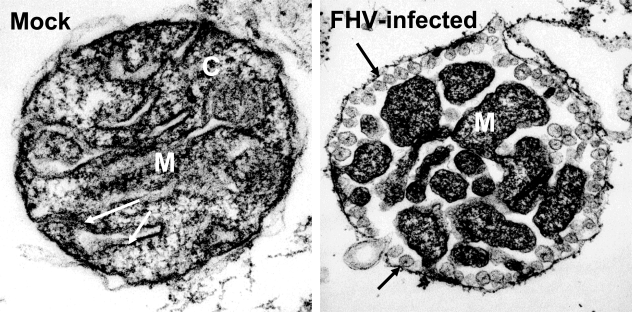
Membrane alterations induced by positive-sense RNA virus replication. Transmission electron micrographs of mitochondria isolated from mock (left) and Flock House virus-infected (right) *Drosophila* cells. Note the normal matrix (M) and cristae (C) in mock mitochondria, whereas the matrix is compacted in mitochondria from infected cells. Furthermore, the outer mitochondrial membrane is studded with spherules (black arrows), which represent viral RNA replication factories.

Positive-sense RNA viruses induce a number of distinctive membrane structures, which include the “membranous webs” of hepatitis C virus [24], the clustered vesicles of picornaviruses [27,29], the double-membrane vesicles of coronaviruses [15,20,21], flaviviruses [22,23], and arteriviruses [15,16], and the spherule-like cytopathic vacuoles of togaviruses that resemble cellular multivesicular bodies [30–32]. One virus whose membrane-induced structures have been examined in detail is Flock House virus, which induces spherule-like invaginations within the outer mitochondrial membrane (Figure 2). These structures, termed virus-induced “mini-organelles”, have been examined using tomographic electron microscopy to provide unprecedented detail and develop three-dimensional models of viral RNA replication complexes [38]. Similar tomographic models have also recently been described for dengue virus [23] and SARS-coronavirus [20], two positive-sense RNA viruses that extensively modify endoplasmic reticulum membranes.

**Figure 3.**
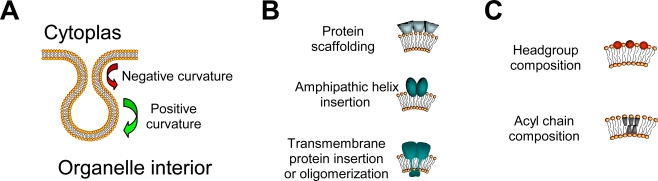
Schematics of membrane curvature necessary to form virus-induced membrane structures **(A)**, and potential protein **(B)** or lipid **(C)** modification that may induce membrane curvature.

The structural changes that must occur within lipid bilayers to produce the virus-induced alterations in intracellular membranes are substantial. For example, the outer mitochondrial membrane invaginations induced by Flock House virus [38] (see also Figure 2) and the endoplasmic reticulum changes induced by dengue virus [23] and SARS-coronavirus [20] require regions of negative and positive curvature, as depicted schematically in Figure 3A. Membrane curvature can be induced by both protein and lipid modifications [77], which are depicted in Figures 3B and 3C, respectively. Protein modifications that induce membrane curvature include internal and external protein scaffolding by peripheral membrane proteins, such as cellular clathrin and calveolin, insertion of proteins with amphipathic helices, such as cellular amphiphysin and endophilins, and insertion or oligomerization of transmembrane proteins, for which there are numerous cellular examples. For positive-sense RNA viruses, replicase proteins with amphipathic helices [44,78,79] or transmembrane domains [68,70] with demonstrated membrane-binding characteristics have also been described. In addition, expression of specific viral proteins alone, in the absence of active viral RNA replication, can frequently induce intracellular membrane structures reminiscent of those induced by viral infections [16,24,29].

An alternative mechanism to induce membrane curvature is modification of lipid structure, either through changes in the polar headgroup or acyl chain composition. For example, lysophospholipids, which contain only one acyl chain per phospholipid molecule, and special lipids such as cholesterol or cardiolipin, which has four acyl chains attached to diphosphatidylglycerol, can have profound impacts on membrane curvature and plasticity [80]. Acyl chain length and saturation can also impact membrane curvature [81]. Although the currently available data on the potential phospholipid alterations that are induced by and/or required for positive-sense RNA virus replication are limited, there are suggestive reports that specific and functionally important changes do occur. Brome mosaic virus RNA replication is suppressed in cells that lack Δ9 fatty acid desaturase and hence contain reduced levels of unsaturated fatty acids [49], Flock House virus induces a preferential increase in lipid molecules with longer and unsaturated acyl chains [54], and West Nile virus redistributes cholesterol to sites of active viral replication [56]. We anticipate that continued advances in cell fractionation, membrane isolation, and lipidomics techniques will begin to address this substantial gap in our understanding of positive-sense RNA virus biology.

**Figure 4.**
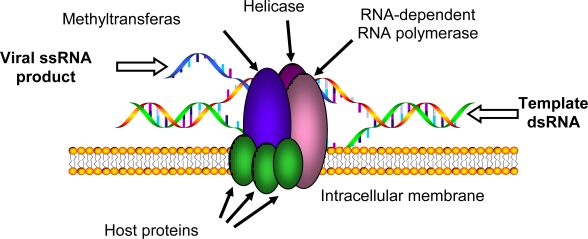
Schematic of positive-sense RNA virus replication complex. Virus-encoded proteins with known or hypothesized enzymatic functions are labeled at the top.

Regardless of the mechanism whereby membrane curvature occurs, the function of these curious virus-induced structures remains enigmatic. One interesting possibility is that virus-induced membrane structures shield viral products that have the potential to activate cellular innate immune pathways. The replication of positive-sense RNA viruses involves the formation of dsRNA intermediates that serve as templates for genome amplification (see below, Figure 4). These replication intermediates, or other “foreign” chemical moieties such as 5′ triphosphorylated RNAs, are potent stimuli for inducing innate antiviral immune responses [82]. Since positive-sense RNA viruses replicate almost exclusively within the cytoplasm of cells, they likely employ mechanisms to suppress or modify activation or amplification of these responses. Positive-sense RNA viruses possess multiple mechanisms to evade innate immune system activation, including the production of proteases that cleave, degrade, or inhibit essential innate immune signaling pathway components or the use of viral proteins to sequester viral dsRNA (reviewed in ref [83,84]). It is intriguing to speculate that virus-induced membrane structures may also play a role in sequestering or shielding viral products from the innate immune system, thus providing an additional level of protection.

### 1.3. Lipids and replication complex function

One major goal of positive-sense RNA virus research is the complete isolation, characterization, and de novo synthesis of fully functional RNA replication complexes, a generic example of which is shown schematically in Figure 4. The achievement of this goal has been hampered in part by the essential membrane-bound nature of these complexes. Substantial hurdles include the frequent inability to obtain soluble viral replicase proteins for detailed biochemical or structural studies, the inherent difficulties in interpreting protein co-purification results when detergents are required to solubilize viral RNA replication complexes, and the presence of three different types of molecular structures (proteins, lipids, and nucleic acids) that are all components of the RNA replication complex yet have different chemical characteristics. However, despite these difficulties some progress has been made. For example, *de novo* synthesis of a functional RNA replication complex in cell-free extracts has been accomplished for both poliovirus [85] and several related plant viruses [86], and this important step will have to be achieved for other viruses as well to provide additional systems in which to investigate the cellular components needed to assemble these viral genome production factories.

A substantial focus in the field of positive-sense RNA virus research has been the identification and characterization of the protein components necessary for replication complex function. The impact of lipids on this process has received far less attention in the past, in part due to some of the technical difficulties in lipid research noted above. However, there is both circumstantial and direct evidence that lipids can serve as functional co-factors for viral replicase proteins [18,26,28,40–45]. For example, although individual viruses typically assemble replication complexes on particular intracellular membranes (see Table 1), this specific targeting can be disrupted by altering either virus-encoded targeting signals [26,35] or host membrane-specific signals [71]. Furthermore, these “retargeted” replication complexes retain functional activity, suggesting that any necessary host components are widely present in multiple intracellular membranes. Although the exact lipid composition of intracellular membranes varies between organelles, phospholipids represent a prominent component of all intracellular membranes [76]. Positive-sense RNA virus replication complexes can be recovered from infected cells by density gradient centrifugation and the isolation of membrane fractions, which retain detergent-sensitive enzymatic activity. Although for many viruses this enzymatic activity is limited to primer-independent synthesis of a complementary negative strand, fully functional replication complexes from Flock House virus-infected cells that produce single-stranded positive-sense products have been described [42], where replicase activity is stimulated by specific phospholipids [45]. In addition, phospholipids have also been shown to influence alphavirus replicase protein activity [44,87]. These observations suggest that in addition to the potential structural roles of lipids in positive-sense RNA virus replication complex assembly described above, lipids may also play important functional roles to maximize replication complex activity.

## 2. Conclusions

Despite the axiom that cellular membranes are essential host factors for positive-sense RNA virus replication, the specific role of individual membrane components, and in particular lipids, represents a vastly understudied area of virus biology and pathogenesis. We have discussed three potential structural or functional roles that cellular membrane-resident lipids may play in the assembly and function of positive-sense RNA virus replication complexes. These roles are not mutually exclusive, and it is possible that lipids contribute to several steps in the virus life cycle via multiple mechanisms, some of which we currently recognize, and others that remain to be discovered. The recent increase in targeted and genome-wide screens to identify host factors that impact positive-sense RNA virus replication [54–63], several of which have highlighted the importance of lipid metabolism-associated genes, provides an exciting foundation for these discoveries.

## Figures and Tables

**Table 1.** Examples of diverse intracellular membranes used by positive-sense RNA viruses to assemble functional RNA replication complexes

**Family**	**Virus**	**Membrane(s)**	**References**
*Arteriviridae*	Equine arteritis virus	Endoplasmic reticulum	[15,16]
*Bromoviridae*	Alfalfa mosaic virus Brome mosaic virus	Vacuole Endoplasmic reticulum	[17,18]
*Coronaviridae*	SARS-coronavirus	Endoplasmic reticulum/Golgi	[19–21]
*Flaviviridae*	Hepatitis C virus Dengue virus West Nile virus	Endoplasmic reticulum Endoplasmic reticulum Endoplasmic reticulum/Golgi	[22–24]
*Nodaviridae*	Flock House virus	Mitochondria	[25,26]
*Picornaviridae*	Poliovirus	Endoplasmic reticulum/Golgi	[27–29]
*Togaviridae*	Rubella virus Semliki Forest virus	Lysosomes Endosomes/lysosomes	[30–32]
*Tombusviridae*	Carnation Italian ringspot virus Cucumber necrosis virus Tomato bushy stunt virus	Mitochondria Endoplasmic reticulum Peroxisome	[33–36]
*Tymoviridae*	Turnip yellow mosaic virus	Chloroplast	[37]

## References

[1] Morse SS (1997). The public health threat of emerging viral diseases. J Nutrition.

[2] Gubler DJ (2002). The global emergence/resurgence of arboviral diseases as public health problems. Arch Med Res.

[3] Drake JW, Holland JJ (1999). Mutation rates among RNA viruses. Proc Natl Acad Sci USA.

[4] Pillay D, Zambon M (1998). Antiviral drug resistance. BMJ.

[5] Fox JL (2007). Antivirals become a broader enterprise. Nat Biotechnol.

[6] Ye J, Wang C, Sumpter R, Brown MS, Goldstein JL, Gale M (2003). Disruption of hepatitis C virus RNA replication through inhibition of host protein geranylgeranylation. Proc Natl Acad Sci USA.

[7] Ye J (2007). Reliance of host cholesterol metabolic pathways for the life cycle of hepatitis C virus. PLoS Pathog.

[8] Ikeda M, Kato N (2007). Modulation of host metabolism as a target of new antivirals. Adv Drug Deliv Rev.

[9] Amemiya F, Maekawa S, Itakura Y, Kanayama A, Matsui A, Takano S, Yamaguchi T, Itakura J, Kitamura T, Inoue T, Sakamoto M, Yamauchi K, Okada S, Yamashita A, Sakamoto N, Itoh M, Enomoto N (2008). Targeting lipid metabolism in the treatment of hepatitis C virus infection. J Infect Dis.

[10] Dreher TW, Miller WA (2006). Translational control in positive strand RNA plant viruses. Virology.

[11] Bushell M, Sarnow P (2002). Hijacking the translation apparatus by RNA viruses. J Cell Biol.

[12] Miller S, Krijnse-Locker J (2008). Modification of intracellular membrane structures for virus replication. Nat Rev Microbiol.

[13] Denison MR (2008). Seeking membranes: positive-strand RNA virus replication complexes. PLoS Biol.

[14] Ahlquist P, Noueiry AO, Lee WM, Kushner DB, Dye BT (2003). Host factors in positive-strand RNA virus genome replication. J Virol.

[15] van der Meer Y, van Tol H, Locker JK, Snijder EJ (1998). ORF1a-encoded replicase subunits are involved in the membrane association of the arterivirus replication complex. J Virol.

[16] Posthuma CC, Pedersen KW, Lu Z, Joosten RG, Roos N, Zevenhoven-Dobbe JC, Snijder EJ (2008). Formation of the arterivirus replication/transcription complex: a key role for nonstructural protein 3 in the remodeling of intracellular membranes. J Virol.

[17] van der Heijden MW, Carette JE, Reinhoud PJ, Haegi A, Bol JF (2001). Alfalfa mosaic virus replicase proteins P1 and P2 interact and colocalize at the vacuolar membrane. J Virol.

[18] Schwartz M, Chen J, Janda M, Sullivan M, den Boon J, Ahlquist P (2002). A positive-strand RNA virus replication complex parallels form and function of retrovirus capsids. Mol Cell.

[19] van der Meer Y, Snijder EJ, Dobbe JC, Schleich S, Denison MR, Spaan WJ, Locker JK (1999). Localization of mouse hepatitis virus nonstructural proteins and RNA synthesis indicates a role for late endosomes in viral replication. J Virol.

[20] Knoops K, Kikkert M, Worm SH, Zevenhoven-Dobbe JC, van der Meer Y, Koster AJ, Mommaas AM, Snijder EJ (2008). SARS-coronavirus replication is supported by a reticulovesicular network of modified endoplasmic reticulum. PLoS Biol.

[21] Ulasli M, Verheije MH, de Haan CA, Reggiori F (2010). Qualitative and quantitative ultrastructural analysis of the membrane rearrangements induced by coronavirus. Cell Microbiol.

[22] Westaway EG, Mackenzie JM, Kenney MT, Jones MK, Khromykh AA (1997). Ultrastructure of Kunjin virus-infected cells: colocalization of NS1 and NS3 with double-stranded RNA, and of NS2B with NS3, in virus- induced membrane structures. J Virol.

[23] Welsch S, Miller S, Romero-Brey I, Merz A, Bleck CK, Walther P, Fuller SD, Antony C, Krijnse-Locker J, Bartenschlager R (2009). Composition and three-dimensional architecture of the dengue virus replication and assembly sites. Cell Host Microbe.

[24] Egger D, Wolk B, Gosert R, Bianchi L, Blum HE, Moradpour D, Bienz K (2002). Expression of hepatitis C virus proteins induces distinct membrane alterations including a candidate viral replication complex. J Virol.

[25] Miller DJ, Schwartz MD, Ahlquist P (2001). Flock House virus RNA replicates on outer mitochondrial membranes in *Drosophila* cells. J Virol.

[26] Miller DJ, Schwartz MD, Dye BT, Ahlquist P (2003). Engineered retargeting of viral RNA replication complexes to an alternative intracellular membrane. J Virol.

[27] Schlegel A, Giddings THJ, Ladinsky MS, Kirkegaard K (1996). Cellular origin and ultrastructure of membranes induced during poliovirus infection. J Virol.

[28] Bienz K, Egger D, Pfister T, Troxler M (1992). Structural and functional characterization of the poliovirus replication complex. J Virol.

[29] Suhy DA, Giddings THJ, Kirkegaard K (2000). Remodeling the endoplasmic reticulum by poliovirus infection and by individual viral proteins: an autophagy-like origin for virus-induced vesicles. J Virol.

[30] Magliano D, Marshall JA, Bowden DS, Vardaxis N, Meanger J, Lee JY (1998). Rubella virus replication complexes are virus-modified lysosomes. Virology.

[31] Kujala P, Ikaheimonen A, Ehsani N, Vihinen H, Auvinen P, Kaariainen L (2001). Biogenesis of the Semliki Forest virus RNA replication complex. J Virol.

[32] Froshauer S, Kartenbeck J, Helenius A (1988). Alphavirus RNA replicase is located on the cytoplasmic surface of endosomes and lysosomes. J Cell Biol.

[33] Weber-Lotfi F, Dietrich A, Russo M, Rubino L (2002). Mitochondrial targeting and membrane anchoring of a viral replicase in plant and yeast cells. J Virol.

[34] Turner KA, Sit TL, Callaway AS, Allen NS, Lommel SA (2004). Red clover necrotic mosaic virus replication proteins accumulate at the endoplasmic reticulum. Virology.

[35] Burgyan J, Rubino L, Russo M (1996). The 5′-terminal region of a tombusvirus genome determines the origin of multivesicular bodies. J Gen Virol.

[36] Rubino L, Weber-Lotfi F, Dietrich A, Stussi-Garaud C, Russo M (2001). The open reading frame 1-encoded (‘36K’) protein of Carnation Italian ringspot virus localizes to mitochondria. J Gen Virol.

[37] Prod'homme D, Le Panse S, Drugeon G, Jupin I (2001). Detection and subcellular localization of the turnip yellow mosaic virus 66K replication protein in infected cells. Virology.

[38] Kopek BG, Perkins G, Miller DJ, Ellisman MH, Ahlquist P (2007). Three-dimensional analysis of a viral RNA replication complex reveals a virus-induced mini-organelle. PLoS Biol.

[39] Mas P, Beachy RN (1999). Replication of tobacco mosaic virus on endoplasmic reticulum and role of the cytoskeleton and virus movement protein in intracellular distribution of viral RNA. J Cell Biol.

[40] Barton DJ, Sawicki SG, Sawicki DL (1991). Solubilization and immunoprecipitation of alphavirus replication complexes. J Virol.

[41] Chu PWG, Westaway EG (1992). Molecular and ultrastructural analysis of heavy membrane fractions associated with the replication of Kunjin virus RNA. Arch Virol.

[42] Wu SX, Kaesberg P (1991). Synthesis of template-sense, single-strand Flock House virus RNA in a cell-free replication system. Virology.

[43] van Hemert MJ, van den Worm SH, Knoops K, Mommaas AM, Gorbalenya AE, Snijder EJ (2008). SARS-coronavirus replication/transcription complexes are membrane-protected and need a host factor for activity *in vitro*. PLoS Pathog.

[44] Ahola T, Lampio A, Auvinen P, Kaariainen L (1999). Semliki Forest virus mRNA capping enzyme requires association with anionic membrane phospholipids for activity. EMBO J.

[45] Wu SX, Ahlquist P, Kaesberg P (1992). Active complete *in vitro* replication of nodavirus RNA requires glycerophospholipid. Proc Natl Acad Sci USA.

[46] Guinea R, Carrasco L (1990). Phospholipid biosynthesis and poliovirus genome replication, two coupled phenomena. EMBO J.

[47] Maynell LA, Kirkegaard K, Klymkowsky MK (1992). Inhibition of poliovirus RNA synthesis by brefeldin A. J Virol.

[48] Perez L, Guinea R, Carrasco L (1991). Synthesis of Semliki Forest virus RNA requires continuous lipid synthesis. Virology.

[49] Lee WM, Ishikawa M, Ahlquist P (2001). Mutation of host Δ9 fatty acid desaturase inhibits brome mosaic virus RNA replication between template recognition and RNA synthesis. J Virol.

[50] Kampmueller KM, Miller DJ (2005). The cellular chaperone heat shock protein 90 facilitates Flock House virus RNA replication in *Drosophila* cells. J Virol.

[51] Yang W, Hood BL, Chadwick SL, Liu S, Watkins SC, Luo G, Conrads TP, Wang T (2008). Fatty acid synthase is up-regulated during hepatitis C virus infection and regulates hepatitis C virus entry and production. Hepatology.

[52] Kapadia SB, Chisari FV (2005). Hepatitis C virus RNA replication is regulated by host geranylgeranylation and fatty acids. Proc Natl Acad Sci USA.

[53] Rassmann A, Henke A, Jarasch N, Lottspeich F, Saluz HP, Munder T (2007). The human fatty acid synthase: a new therapeutic target for coxsackievirus B3-induced diseases. Antiviral Res.

[54] Castorena KM, Stapleford KA, Miller DJ (2010). Complementary transcriptomic, lipidomic, and targeted functional genetic analyses in cultured *Drosophila* cells highlight the role of glycerophospholipid metabolism in Flock House virus RNA replication. BMC Genomics.

[55] Cherry S, Kunte A, Wang H, Coyne C, Rawson RB, Perrimon N (2006). COPI activity coupled with fatty acid biosynthesis is required for viral replication. PLoS Pathog.

[56] Mackenzie JM, Khromykh AA, Parton RG (2007). Cholesterol manipulation by West Nile virus perturbs the cellular immune response. Cell Host Microbe.

[57] Panavas T, Serviene E, Brasher J, Nagy PD (2005). Yeast genome-wide screen reveals dissimilar sets of host genes affecting replication of RNA viruses. Proc Natl Acad SciU SA.

[58] Kushner DB, Lindenbach BD, Grdzelishvili VZ, Noueiry AO, Paul SM, Ahlquist P (2003). Systematic, genome-wide identification of host genes affecting replication of a positive-strand RNA virus. Proc Natl Acad Sci USA.

[59] Jiang Y, Serviene E, Gal J, Panavas T, Nagy PD (2006). Identification of essential host factors affecting tombusvirus RNA replication based on the yeast Tet promoters Hughes Collection. J Virol.

[60] Berger KL, Cooper JD, Heaton NS, Yoon R, Oakland TE, Jordan TX, Mateu G, Grakoui A, Randall G (2009). Roles for endocytic trafficking and phosphatidylinositol 4-kinase III alpha in hepatitis C virus replication. Proc Natl Acad Sci USA.

[61] Borawski J, Troke P, Puyang X, Gibaja V, Zhao S, Mickanin C, Leighton-Davies J, Wilson CJ, Myer V, Cornellataracido I, Baryza J, Tallarico J, Joberty G, Bantscheff M, Schirle M, Bouwmeester T, Mathy JE, Lin K, Compton T, Labow M, Wiedmann B, Gaither LA (2009). Class III phosphatidylinositol 4-kinase α and β are novel host factor regulators of hepatitis C virus replication. J Virol.

[62] Tai AW, Benita Y, Peng LF, Kim SS, Sakamoto N, Xavier RJ, Chung RT (2009). A functional genomic screen identifies cellular cofactors of hepatitis C virus replication. Cell Host Microbe.

[63] Vaillancourt FH, Pilote L, Cartier M, Lippens J, Liuzzi M, Bethell RC, Cordingley MG, Kukolj G (2009). Identification of a lipid kinase as a host factor involved in hepatitis C virus RNA replication. Virology.

[64] Annamalai P, Rofail F, Demason DA, Rao AL (2008). Replication-coupled packaging mechanism in positive-strand RNA viruses: synchronized coexpression of functional multigenome RNA components of an animal and a plant virus in *Nicotiana benthamiana* cells by agroinfiltration. J Virol.

[65] Venter PA, Schneemann A (2007). Assembly of two independent populations of Flock House virus particles with distinct RNA packaging characteristics in the same cell. J Virol.

[66] Nugent CI, Johnson KL, Sarnow P, Kirkegaard K (1999). Functional coupling between replication and packaging of poliovirus replicon RNA. J Virol.

[67] Den Boon JA, Chen J, Ahlquist P (2001). Identification of sequences in brome mosaic virus replicase protein 1a that mediate association with endoplasmic reticulum membranes. J Virol.

[68] Miller DJ, Ahlquist P (2002). Flock House virus RNA polymerase is a transmembrane protein with amino-terminal sequences sufficient for mitochondrial localization and membrane insertion. J Virol.

[69] Schaad MC, Jensen PE, Carrington JC (1997). Formation of plant RNA virus replication complexes on membranes: role of an endoplasmic reticulum-targeted viral protein. EMBO J.

[70] Schmidt-Mende J, Bieck E, Hügle T, Penin F, Rice CM, Blum HE, Moradpour D (2001). Determinants for membrane association of the hepatitis C virus RNA-dependent RNA polymerase. J Biol Chem.

[71] Jonczyk M, Pathak KB, Sharma M, Nagy PD (2007). Exploiting alternative subcellular location for replication: tombusvirus replication switches to the endoplasmic reticulum in the absence of peroxisomes. Virology.

[72] Nishikiori M, Dohi K, Mori M, Meshi T, Naito S, Ishikawa M (2006). Membrane-bound tomato mosaic virus replication proteins participate in RNA synthesis and are associated with host proteins in a pattern distinct from those that are not membrane bound. J Virol.

[73] Pathak KB, Sasvari Z, Nagy PD (2008). The host Pex19p plays a role in peroxisomal localization of tombusvirus replication proteins. Virology.

[74] Stapleford KA, Rapaport D, Miller DJ (2009). Mitochondrion-enriched anionic phospholipids facilitate Flock House virus RNA polymerase membrane association. J Virol.

[75] Zinser E, Daum G (1995). Isolation and biochemical characterization of organelles from the yeast *Saccharomyces cerevisiae*. Yeast.

[76] van Meer G, Voelker DR, Feigenson GW (2008). Membrane lipids: where they are and how they behave. Nat Rev Mol Cell Biol.

[77] McMahon HT, Gallop JL (2005). Membrane curvature and mechanisms of dynamic cell membrane remodeling. Nature.

[78] Elazar M, Cheong KH, Liu P, Greenberg HB, Rice CM, Glenn JS (2003). Amphipathic helix-dependent localization of NS5A mediates hepatitis C virus RNA replication. J Virol.

[79] Liu L, Westler WM, den Boon JA, Wang X, Diaz A, Steinberg HA, Ahlquist P (2009). An amphipathic α-helix controls multiple roles of brome mosaic virus protein 1a in RNA replication complex assembly and function. PLoS Pathog.

[80] Fuller N, Rand RP (2001). The influence of lysolipids on the spontaneous curvature and bending elasticity of phospholipid membranes. Biophys J.

[81] Szule JA, Fuller NL, Rand RP (2002). The effects of acyl chain length and saturation of diacylglycerols and phosphatidylcholines on membrane monolayer curvature. Biophys J.

[82] Pichlmair A, Reis e Sousa C (2007). Innate recognition of viruses. Immunity.

[83] Suthar MS, Gale M, Owen DM (2009). Evasion and disruption of innate immune signalling by hepatitis C and West Nile viruses. Cell Microbiol.

[84] Bowie AG, Unterholzner L (2008). Viral evasion and subversion of pattern-recognition receptor signalling. Nat Rev Immunol.

[85] Molla A, Paul AV, Wimmer E (1991). Cell-free, de novo synthesis of poliovirus. Science.

[86] Komoda K, Naito S, Ishikawa M (2004). Replication of plant RNA virus genomes in a cell-free extract of evacuolated plant protoplasts. Proc Natl Acad Sci USA.

[87] Saito K, Nishijima M, Kuge O (2006). Phosphatidylserine is involved in gene expression from Sindbis virus subgenomic promoter. Biochem Biophys Res Comm.

